# In the Shade of Ygdrasil

**Published:** 1903-05

**Authors:** Frederick Peterson


### In the Shade of Ygdrasil.[1]

By FREDERICK PETERSON.

### HEREDITY.

I meet upon the woodland ways
At morn a lady fair;
Adown her slender shoulders strays
Her raven hair;

And none who looks into her eyes
Can fail to feel and know
That in this conscious clay there lies
Some soul aglow.

But I, who meet her oft about
The woods in morning song,
I see behind her far stretch out
A ghostly throng—

A priest, a prince, a lord, a maid,
Faces of grief and sin,
A high-born lady and a jade,
A harlequin—

Two lines of ghosts in masquerade,
Who push her where they will,
As if it were the wind that swayed
A daffodil.

She sings, she weeps, she smiles, she sighs,
Looks cruel, sweet or base;
The features of her fathers rise
And haunt her face.

As if it were the wind that swayed
Some stately daffodil,
Upon her face they masquerade
And work their will.

### ENVIRONMENT.

High up around the mountain rock
Wild sweep the lightning and the storm;
The spruce grows firm against their shock,
Stunted and gnarled and rude of form,
With twisted roots that interlock.

But by the rivulet far below,
Up from the rich dark loam and drift,
Where storms come not and winds are slow,
Behold the stately willow lift
And sway long branches to and fro!

1. From the proceedings of the Charaka Club, Vol. I.

